# Tandem cross enyne metathesis (CEYM)–intramolecular Diels–Alder reaction (IMDAR). An easy entry to linear bicyclic scaffolds

**DOI:** 10.3762/bjoc.11.161

**Published:** 2015-08-25

**Authors:** Javier Miró, María Sánchez-Roselló, Álvaro Sanz, Fernando Rabasa, Carlos del Pozo, Santos Fustero

**Affiliations:** 1 Departamento de Química Orgánica, Universidad de Valencia, E-46100 Burjassot, Spain; 2 Laboratorio de Moléculas Orgánicas, Centro de Investigación Príncipe Felipe, E-46012 Valencia, Spain

**Keywords:** bicyclic frameworks, cross enyne metathesis, Diels–Alder reaction, tandem reaction

## Abstract

A new tandem cross enyne metathesis (CEYM)–intramolecular Diels–Alder reaction (IMDAR) has been carried out. It involves conjugated ketones, esters or amides bearing a remote olefin and aromatic alkynes as the starting materials. The overall process enables the preparation of a small family of linear bicyclic scaffolds in a very simple manner with moderate to good levels of diastereoselectivity. This methodology constitutes one of the few examples that employ olefins differently than ethylene in tandem CEYM–IMDAR protocols.

## Introduction

Among all metathetic processes, the enyne metathesis reaction has received significant attention as an attractive and frequently used synthetic tool in organic synthesis [1–7]. It is an atom economical process that combines alkene and alkyne moieties to generate conjugated 1,3-dienes under mild conditions. These 1,3-dienes are versatile building blocks suitable for further non-metathetic transformations, either in a step-wise or a tandem fashion. Thus, the enyne metathesis methodology has become a powerful tool for the generation of carbon–carbon bonds, expanding the utility of metathesis processes beyond olefinic substrates [8–9].

The inherent tandem nature of enyne metathesis is particularly appealing in its combination with the Diels–Alder reaction. This tandem protocol is well suited for addressing a broad range of complex molecules since multiple carbon–carbon bonds can be generated in a single operation, therefore increasing molecular complexity in a quite simple manner [10].

While examples of ring-closing enyne metathesis (RCEYM) reactions are widespread in the literature [11], the development of the intermolecular version, i.e., the cross enyne metathesis (CEYM), lagged behind probably due to difficulties in controlling the stereoselectivity in the newly formed double bond leading to the formation of mixtures of *E* and *Z*-isomers. These inherent selectivity problems are absent when the olefin counterpart is the ethylene unit, which explains why most of the reported examples that combine a CEYM reaction with a Diels–Alder cycloaddition in a tandem manner involve the use of ethylene as the olefin partner either by employing an internal source of it or by bubbling it into the reaction mixture. This strategy allowed for the synthesis of a wide variety of natural and non-natural products in the last decade [12–22].

**Scheme 1:**
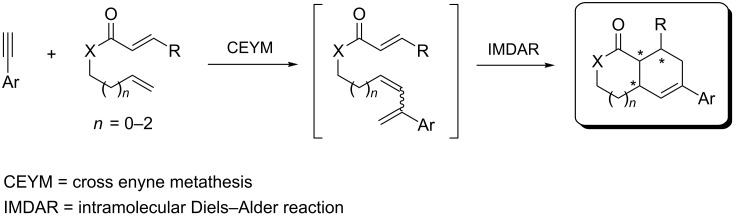
Tandem cross enyne metathesis–intramolecular Diels–Alder reaction.

The use of olefins other than ethylene in CEYM-Diels–Alder tandem protocols is very scarce. The first example was reported in 2005 by combining Baylis–Hillman adducts with alkynes in the presence of second generation Hoveyda–Grubbs catalyst [23]. After the initial formation of the trienic unit, an intramolecular Diels–Alder reaction (IMDAR) rendered highly functionalized bicyclic derivatives in a very efficient manner. More recently, a multicomponent CEYM–intermolecular hetero-Diels–Alder reaction involving alkynes, ethyl glyoxalate and ethyl vinyl ether was described for the preparation of 2,3-dihydropyrans [24–25]. Additionally, a tandem CEYM–IMDAR reaction in combination with a final aromatization step was employed for the synthesis of biaryl derivatives [26]. Herein, a new example of this tandem protocol CEYM–IMDAR with alkynes and α,ω-dienes as starting materials is reported, which will give access to a new family of linear bicyclic carbo- and heterocyclic scaffolds. We envisioned that the initial CEYM would occur in the electronically neutral olefin to generate the corresponding triene intermediate, which would evolve under the reaction conditions through the cycloaddition event to render the final products (Scheme 1).

## Results and Discussion

The use of 1,5-, 1,6- and 1,7-dienes in cross metathesis-type transformations is not trivial since chemoselectivity issues can arise. It is well known that electronically deficient olefins should undergo metathesis in a slow rate based on the model developed by Grubbs and coworkers that classifies olefins and predicts their reactivity in CM reactions [27]. We anticipated that, according to these studies, in substrates bearing two different olefin units one being an α,β-unsaturated moiety, the tandem CM–IMDAR protocol would initiate on the electronically neutral olefin. Furthermore, those dienes could undergo an intramolecular cyclization (RCM) promoted by the ruthenium carbene that would compete with the desired intermolecular CM process.

In order to prove our assumptions, phenylacetylene (**1a**) and conjugated ester **2a** were employed as model substrates to study the tandem protocol. The results obtained in the optimization process are summarized in Table 1.

**Table 1:** Optimization of the tandem CEYM–IMDAR.

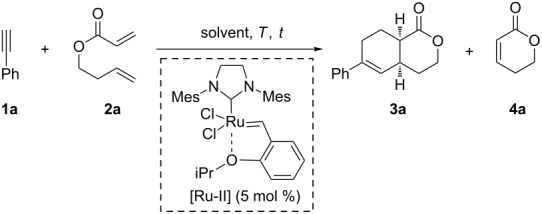						

entry	solvent	*T* (°C)	**1a**:**2a**	*t* (h)	additive	% yield **3a**^a^

1	toluene	90	1:3	6	–	25
2	toluene	90	1:3	24	–	37
3	toluene	90	1:3	48	–	57
4	toluene	90	1:3	72	–	39
5	toluene	90	1:1	48	–	36
6	toluene	90	1:5	48	–	30
7	toluene	110	1:3	48	–	52
8	toluene	140	1:3	48	–	37
9	DCM	60	1:3	48	–	25
10	C_6_H_5_CF_3_	90	1:3	48	–	60
11	toluene	90	1:3	48	Ti(OiPr)_4_^b^	38
12	toluene	90	1:3	48	BF_3_·OEt_2_^b^	15
13	toluene	90	1:3	48	thiourea^c^	49
14	toluene	90	1:3	48	BQ^b,d^	37

^a^Isolated yield after column chromatography. Variable amounts of **4a** were observed in all cases, but never exceeded 15% (based on **2a**). Some unreacted **2a** was also detected in all cases; ^b^5 mol %; ^c^1 mol %; ^d^benzoquinone.

The first attempt to carry out the projected tandem protocol was performed by heating 1.0 equiv of phenylacetylene (**1a**) and 3.0 equiv of diolefinic ester **2a** in toluene in the presence of second generation Hoveyda–Grubbs catalyst [Ru-II]. After 6 hours at 90 °C, bicyclic lactone **3a** was obtained in 25% yield (Table 1, entry 1), together with lactone **4a** (15%, arising from the ring closing metathesis (RCM) of **2a**), and unreacted **2a**. The isolated yield of **3a** was improved to 57% by increasing the reaction time to 48 hours (Table 1, entries 2 and 3). An extended reaction time (72 h) led to a drop in the final yield (Table 1, entry 4). In all cases variable amounts of **4a**, which never exceeded 15%, and unreacted **2a** were detected in the crude mixture. On the other hand, it is worth noting that although compound **4a** can be considered as a good dienophile, its intermolecular Diels–Alder reaction with the triene intermediate formed after the initial CEYM was not observed. This fact, together with the successful formation of the desired bicycle **3a**, indicates that the CEYM between **1a** and **2a** is faster than the RCM of **2a**, and also that the intramolecular Diels–Alder process is more favoured once the triene unit is formed.

Different ratios of substrates **1a**:**2a** did not improve the efficiency of the process (Table 1, entries 5 and 6). Likewise, higher temperatures afforded comparable yields of product **3a** (Table 1, entries 7 and 8). When the reaction was performed in DCM only 25% of **3a** were isolated, while the use of trifluorotoluene as solvent afforded the best yield (60%) of the tandem process (Table 1, entries 9 and 10).

The use of Lewis acids as co-catalysts was also tested although the efficiency of the process did not improve neither with Ti(OiPr)_4_ nor with BF_3_·OEt_2_ (Table 1, entries 11 and 12). Alternatively, thiourea derivatives have proven to be very effective hydrogen-bonding catalysts for Diels–Alder reactions [28]. However, in our case no influence was observed when the reaction was performed in the presence of diaryl thioureas (Table 1, entry 13). Finally, the use of benzoquinone (BQ) as an additive, which has been reported to suppress the formation of byproducts in enyne metathesis protocols [29], was also unsuccessful in the present case (Table 1, entry 14).

It is noteworthy that compound **3a** was always obtained as a single diastereoisomer showing a *cis* fusion between the two cycles [30].

Next, the optimized conditions (heating at 90 °C for 48 h in the presence of Ru-II catalyst) were applied to other aromatic alkynes **1** and dienes **2**, affording a new family of linear carbocycles and heterocycles **3** in moderate yields (Table 2).

**Table 2:** Scope of the tandem CEYM-IMDAR protocol.

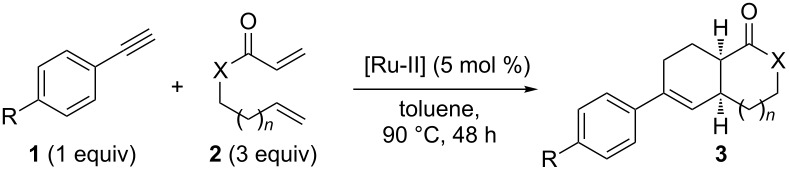			

entry	**1** (R)	**2**	**3** (yield)^a^

1	**1a** (H)	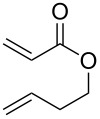 **2a**	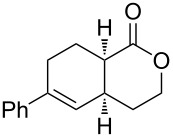 **3a** (57%)
2	**1a** (H)	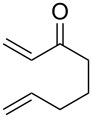 **2b**	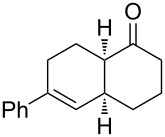 **3b** (38%)
3	**1a** (H)	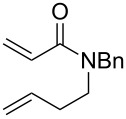 **2c**	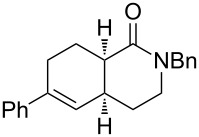 **3c** (62%)
4	**1b** (F)	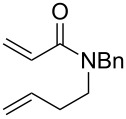 **2c**	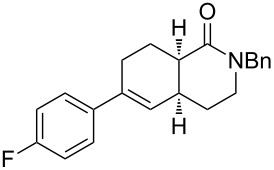 **3d** (45%)
5	**1c** (OMe)	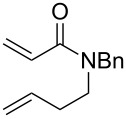 **2c**	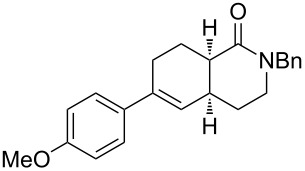 **3e** (35%)
6	**1a** (H)	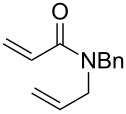 **2d**	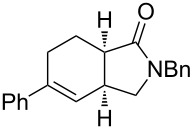 **3f** (50%)
7	**1a** (H)	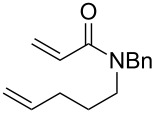 **2e**	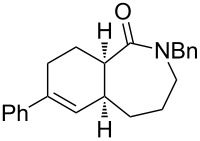 **3g** (44%)

^a^Isolated yields after column chromatography. All final products **3** were obtained as single diastereoisomers.

Bicyclic lactone **3a**, ketone **3b** and lactam **3c** were obtained in moderate yields following the tandem CEYM-IMDAR protocol (Table 2, entries 1–3). Comparable yields were obtained with either electron-donating or electron-withdrawing substituents in the starting alkyne **1** (Table 2, entries 4 and 5). In addition, 5- and 7-membered bicyclic lactams **3f** and **3g** were also synthesized in moderate yields (Table 2, entries 6 and 7). Again, all bicycles **3** were obtained as single diastereoisomers, assuming the same *cis*-stereochemistry as in compound **3a** [30].

**Scheme 2:**
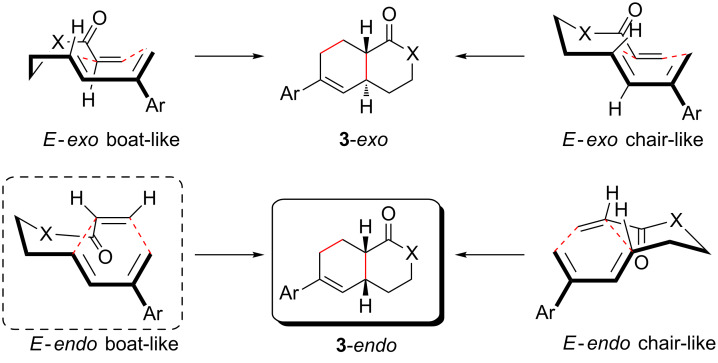
Stereochemical outcome of the IMDAR.

Although it was not possible to isolate the intermediate trienes formed after the initial CEYM under the reaction conditions, they should be formed as a mixture of *E*/*Z* diastereoisomers. We would expect that only the *E*-isomer possesses the adequate disposition to undergo the IMDAR, while the *Z*-isomer would not cyclize. However, since this *Z*-isomer was not detected after 48 h, it was assumed that this triene intermediate decomposes under the reaction conditions or, alternatively, it undergoes an RCM to render compounds **4** (Table 1) and only the final products arising from the *E*-isomer were observed. Moreover, the IMDAR of dienes and dienophiles linked by ester or amide tethers was theoretically studied [31]. These studies indicated that *endo* geometries are favoured over *exo* ones and also that boat-like conformations are preferred over chair-like ones. These studies accurately correlated with the experimental results observed in these types of cyclizations [32–34]. The preference of the boat-like transition state was ascribed to the co-planarity of the carbonyl group during the cycloaddition, being maximized in the *E*-*endo* boat-like transition state leading to the formation of the *cis*-cycloadduct **3**-*endo* (Scheme 2).

**Scheme 3:**
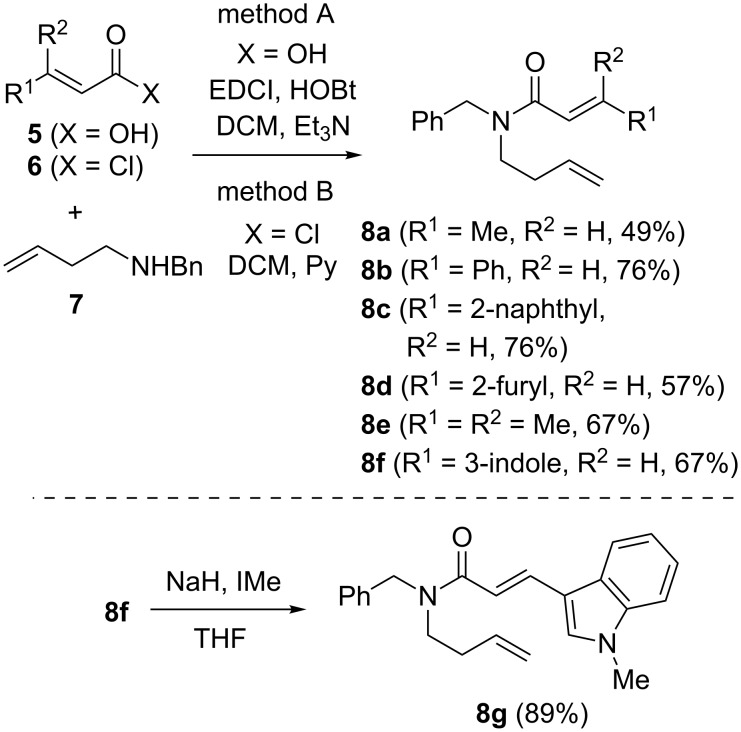
Preparation of starting materials **8**.

The tandem protocol was next extended to substituted dienes **8**. These substrates were assembled by condensation of homoallyl benzylamine **7** with carboxylic acids **5** (method A) or acyl chlorides **6** (method B) under standard conditions (Scheme 3). Since the basic indole nitrogen in substrate **8f** could interfere with the ruthenium catalyst, it was *N*-methylated to render compound **8g**.

With substrates **8** in hand, they were subjected to the optimized conditions of the tandem CEYM–IMDAR protocol. The results of these tandem reactions are depicted in Table 3.

**Table 3:** Extending the scope of the tandem CEYM–IMDAR protocol to amides **8**.

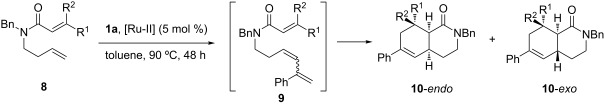				

entry	**8**	**9**	**10**	% yield **10**^a^ (*endo*: *exo*)^b^

1	**8a**	–^c^	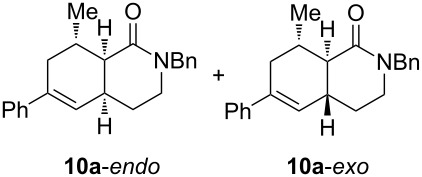	85 (93:7)
2	**8b**	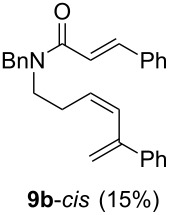	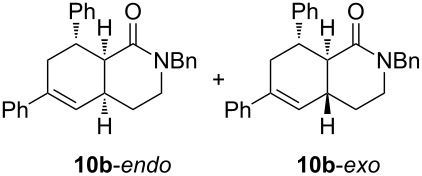	78 (53:47)
3	**8c**	–^c^	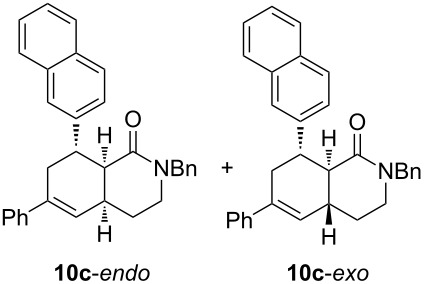	47 (66:34)
4	**8d**	–^c^	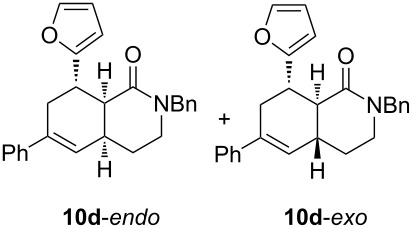	68 (72:28)
5	**8e**	–^c^	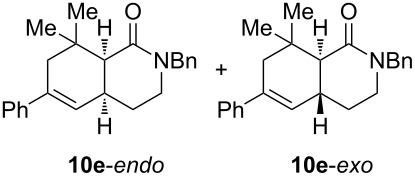	25 (77:23)
6	**8g**	–^c^	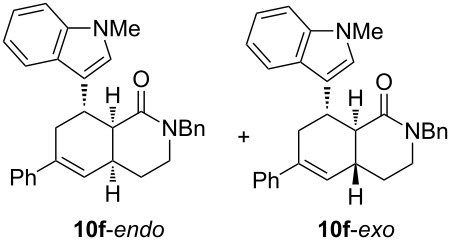	33 (50:50)

^a^Isolated yields after column chromatography; ^b^diasteroisomeric ratio determined by ^1^H NMR; ^c^not observed;

Diolefinic substrate **8a** underwent the tandem sequence in excellent yield (85%) to afford the *endo* isomer **10a**-*endo* as the major product together with a small amount of the *exo* isomer **10a**-*exo* (Table 3, entry 1). On the other hand, compound **8b** bearing a phenyl substituent at the α-olefinic carbon gave an almost equimolecular but separable mixture of bicycles **10b**-*endo* and **10b**-*exo* (78% overall yield). In this case, a small amount of triene intermediate **9b**-*cis* (15% yield) was also isolated, which was in agreement with our previous assumption that the *cis*-triene does not undergo the IMDAR.

Diolefinic amides **8c** and **8d** bearing the 2-naphthyl and 2-furyl substituents, respectively, rendered the corresponding bicyclic products **10c** and **10d** in acceptable yields (47 and 68%) and moderate diastereoselectivity (Table 3, entries 3 and 4). The use of a trisubstituted olefin as the starting material (**8e**) caused a significant drop of the final yield, probably due to steric reasons (Table 3, entry 5). Finally, the indole-containing derivative **8g** gave an equimolecular but separable mixture of adducts **10f**-*endo* and **10f**-*exo* in moderate yield (Table 3, entry 6).

It can be assumed that in these cases, the *E*-*exo* boat-like transition state is also in operation (see Scheme 2), which gives rise to the diastereoisomeric *endo*/*exo* mixtures.

**Figure 1:**
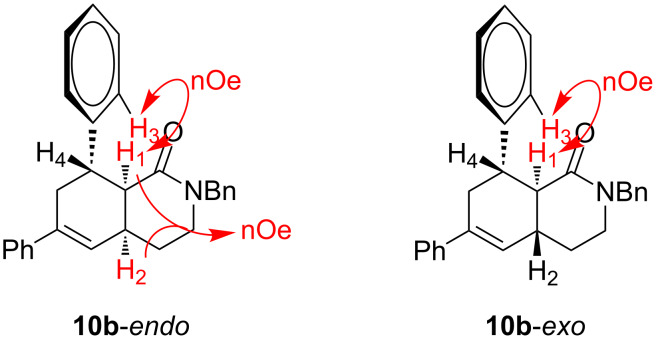
Determination of the relative stereochemistry on compounds **10b**.

The relative stereochemistry of the final products **10** was determined on compounds **10b**-*endo* and **10b**-*exo*. After chromatographic separation, NOESY experiments indicated that **10b**-*endo* shows two nOe correlations: one between H^1^ and H^2^ (which indicates the *cis*-fusion of the two cycles) and another one between H^1^ and the aromatic proton H^3^. These two nOe interactions, together with the absence of a correlation between H^1^ and H^4^ indicated that the phenyl ring and H^1^ display a *cis* relationship. Additionally, compound **10b**-*exo* only showed an nOe correlation between H^1^ and H^3^ (Figure 1). For the rest of compounds **10**, an analogous stereochemical outcome was assumed.

## Conclusion

In conclusion, a new tandem CEYM–IMDAR involving aromatic alkynes and dienes bearing two electronically different olefin moieties is described. Non-substituted substrates **2** are good partners in the tandem protocol affording linear bicyclic derivatives **3** as single diastereoisomers. The IMDAR takes place with complete *endo* selectivity, by means of an *endo* boat-like transition state. The use of substrates **8** with increased substitution at the β-olefinic carbon provides the formation of final products **10** as mixtures of *endo*/*exo* diastereoisomers, indicating that an *exo* boat-like transition state is also in operation in this case. It is noteworthy that this is one of the few examples of this tandem protocol that employs olefins other than ethylene.

## Experimental

**General procedure for the tandem protocol.** A solution of Hoveyda–Grubbs 2nd generation (5 mol %), diene **2** or **8** (3.0 equiv) and alkyne **1** (0.5 mmol) in dry toluene 0.05 M was heated at 90 °C in a sealed tube. The reaction mixture was stirred at this temperature for 48 h. The solvents were then removed under reduced pressure and the crude mixture was purified by flash chromatography in *n*-hexanes/ethyl acetate.

(4aR*,8aS*)-6-Phenyl-3,4,4a,7,8,8a-hexahydro-1*H*-isochromen-1-one (3a). Following the general procedure described above, **3a** was obtained in 57% yield as a brown oil. ^1^H NMR (CDCl_3_, 300 MHz) δ 1.68–1.87 (m, 2H), 2.00–2.10 (m, 1H), 2.25–2.36 (m, 2H), 2.41–2.53 (m, 1H), 2.75–2.85 (m, 2H), 4.22 (dd, *J*_1_ = 6.0 Hz, *J*_2_ = 4.5 Hz, 2H), 5.81–5.83 (m, 1H), 7.13–7.29 (m, 5H); ^13^C NMR (CDCl3, 75.5 MHz) δ 24.1, 24.7, 28.5, 32.5, 38.9, 67.3, 124.8, 125.1, 127.3, 128.3, 139.4, 141.2, 173.4; HRMS (ES): [M + 1]^+^ calcd for C_15_H_17_O_2_, 229.1223; found, 229.1233.

## Supporting Information

File 1 Experimental and analytical data.
