# Supplementary material for: Tandem cross enyne metathesis (CEYM)–intramolecular Diels–Alder reaction (IMDAR). An easy entry to linear bicyclic scaffolds
Source: Beilstein J Org Chem. 2015 Aug 25;11:1486–93. doi: 10.3762/bjoc.11.161 (PMC4578421; doi:10.3762/bjoc.11.161)
Supplement: File 1 — Experimental and analytical data. [file Beilstein_J_Org_Chem-11-1486-s001.pdf]

**Supporting Information**

**for**

# Tandem cross enyne metathesis (CEYM)–intramolecular Diels–Alder reaction (IMDAR). An easy entry to linear bicyclic scaffolds

Javier Miró[1], María Sánchez-Roselló[1,2], Álvaro Sanz[1], Fernando Rabasa[1], Carlos del Pozo*[,1] and Santos Fustero*[,1,2]

Address: [1]Departamento de Química Orgánica, Universidad de Valencia, E-46100 Burjassot, Spain and [2]Laboratorio de Moléculas Orgánicas, Centro de Investigación Príncipe Felipe, E-46012 Valencia, Spain

Email: Santos Fustero - santos.fustero@uv.es, Carlos del Pozo - carlos.pozo@uv.es

*Corresponding author

## Experimental and analytical data

**General experimental methods.** Reactions were carried out under argon atmosphere unless otherwise indicated. The solvents were purified prior to use: THF, diethyl ether and toluene were distilled from sodium/benzophenone, dichloromethane and acetonitrile were distilled from calcium hydride. The reactions were monitored with the aid of thin-layer chromatography (TLC) on 0.25 mm precoated silica gel plates. Visualization was carried out with UV light and aqueous ceric ammonium molybdate solution or potassium permanganate stain. Flash column chromatography

was performed with the indicated solvents on silica gel 60 (particle size 0.040–0.063 mm). $^{1}H$ and $^{13}C$ NMR spectra were recorded on a 300 and a 500 MHz spectrometer, respectively. Chemical shifts are given in ppm (δ), with reference to the residual proton resonances of the solvents. Coupling constants (*J*) are given in Hertz (Hz). The letters m, s, d, t, and q stand for multiplet, singlet, doublet, triplet and quartet, respectively. The letters br indicate that the signal is broad. Starting dienes **2a** [1], **2b** [2], **2c** [3], **2d** [2] and **2e** [4] were previously described.

**Preparation of starting substrates 8, method A.** Homoallylbenzylamine **7** (1.0 mmol) was added to a mixture of the corresponding carboxylic acid **5** (1.0 mmol), EDCI (1.5 mmol), HOBt (2.0 mmol) and triethylamine (2.0 mmol) in DCM (0.10 M). The mixture was stirred overnight at rt. The reaction mixture was then hydrolysed with $H_2O$ (20 mL) and extracted with DCM (3 × 15 mL). Collected organic fractions were dried over $Na_2SO_4$ and, after removal of the solvents the crude reaction mixture was purified by flash chromatography.

**Method B**. Homoallyl benzyl amine **7** (2.3 mmol) was dissolved in DCM (3.6 mL) and pyridine (3.0 mmol) and cooled to 0 °C. A solution of the corresponding acyl chloride **6** (3.0 mmol) in DCM (1.0 mL) was then syringed dropwise into the reaction. The resulting reaction mixture was stirred at room temperature for 3 hours. The reaction was then diluted with DCM (10 mL), quenched with sat $NaHCO_3$ (20 mL), extracted with DCM (3 × 20 mL), dried over $Na_2SO_4$, filtered, and concentrated in vacuum. The crude product was purified through flash chromatography to afford the title compound.

**(*E*)-*N*-Benzyl-*N*-(3-butenyl)-2-butenamide (8a)**

Amide **8a** was obtained in 49% yield (colourless oil) from (*E*)-2-butenoic acid **5a** as a mixture of rotamers following method A. $^{1}H$-NMR ($CDCl_3$, 300 MHz): δ 1.84 (d, *J* =

6.6 Hz, 3/2H), 1.93 (d, *J* = 6.6 Hz, 3/2H), 2.28-2.39 (m, 2H), 3.36 (t, *J* = 7.5 Hz, 1H), 3.49 (t, *J* = 7.5 Hz, 1H), 4.61-4.71 (m, 2H), 5.01-5.11 (m, 2H), 5.68-5.87 (m, 1H), 6.17 (d, *J* = 15.3 Hz, 1/2H), 6.31 (d, *J* = 14.7 Hz, 1/2H), 6.93-7.09 (m, 1H), 7.19-7.37 (m, 5H); $^{13}$C-NMR ($CDCl_3$, 75.5 MHz): δ 18.1, 18.2, 32.1, 33.4, 46.0, 46.5, 49.0, 51.2, 116.5, 117.3, 121.5, 121.7, 126.3, 127.2, 127.4, 127.9, 128.4, 128.7, 134.2, 135.4, 137.1, 137.7, 142.1, 142.3, 166.5, 167.0; HRMS (ES) calc. for ($M^+$+1) $C_{15}H_{20}NO$: 230.1539; found: 230.1545.

**(*E*)-*N*-Benzyl-*N*-(3-butenyl)cinnamamide (8b)**

Amide **8b** was obtained in 76% yield (colourless oil) from (*E*)-cinnamic acid **5b** as a mixture of rotamers following method A. $^{1}$H-NMR ($CDCl_3$, 300 MHz): δ 2.26-2.38 (m, 2H), 3.40 (t, *J* = 7.4 Hz, 1H), 3.51 (t, *J* = 7.4 Hz, 1H), 4.66 (d, *J* = 16.2 Hz, 2H), 4.98-5.08 (m, 2H), 5.66-5.84 (m, 1H), 6.78 (d, *J* = 15.6 Hz, 1/2H), 6.88 (d, *J* = 15.3 Hz, 1/2H), 7.18-7.38 (m, 9H), 7.48-7.50 (m, 1H), 7.76 (t, *J* = 14.9 Hz, 1H); $^{13}$C-NMR ($CDCl_3$, 75.5 MHz): δ 31.9, 33.4, 46.2, 46.5, 49.1, 51.2, 116.5, 117.0, 117.3, 117.4, 126.1, 127.1, 127.4, 127.5, 127.8, 128.3, 128.4, 128.6, 128.6, 129.3, 129.4, 134.0, 134.9, 135.0, 135.1, 136.9, 137.4, 142.8, 142.9, 166.2, 166.6; HRMS (ES) calc. for ($M^+$+1) $C_{20}H_{22}NO$: 292.1696; found: 292.1703.

**(*E*)-*N*-Benzyl-*N*-(3-butenyl)-3-(2-naphthyl)acrylamide (8c)**

Amide **8c** was obtained in 76% yield (colourless oil) from (*E*)-3-(2-naphthyl)acrylic acid **5c** as a mixture of rotamers following method A. $^{1}$H-NMR ($CDCl_3$, 300 MHz): δ 2.40-2.43 (m, 2H), 3.51 (t, *J* = 7.5 Hz, 1H), 3.59 (t, *J* = 7.2 Hz, 1H), 4.71-4.78 (m, 2H), 5.04-5.17 (m, 2H), 5.75-5.91 (m, 1H), 6.93 (d, *J* = 15.3 Hz, 1/2H), 7.03 (d, *J* = 15.3 Hz, 1/2H), 7.27-7.40 (m, 5H), 7.47-8.00 (m, 8H); $^{13}$C-NMR ($CDCl_3$, 75.5 MHz): δ 32.2, 33.7, 46.4, 46.8, 49.4, 51.6, 116.8, 117.3, 117.6, 123.5, 123.6, 126.4, 126.5, 126.6, 126.8, 126.9, 127.3, 127.7, 128.1, 128.4, 128.4, 128.5, 128.6, 128.9, 129.3, 129.4,

132.6, 132.7, 133.3, 133.4, 133.9, 134.3, 135.4, 137.2, 137.7, 143.2, 143.3, 166.6, 167.0; HRMS (ES) calc. for ($M^+$+1) $C_{24}H_{24}NO$: 342.1852; found: 342.1851.

**(*E*)-*N*-Benzyl-*N*-(3-butenyl)-3-(2-furyl)acrylamide (8d)**

Amide **8d** was obtained in 57% yield (brown oil) from (*E*)-3-(2-furyl)acrylic acid **5d** as a mixture of rotamers following method A. $^1$H-NMR ($CDCl_3$, 300 MHz): δ 2.40 (t, *J* = 7.2 Hz, 2H), 3.47 (t, *J* = 7.5 Hz, 1H), 3.57 (t, *J* = 7.5 Hz, 1H), 4.71-4.76 (m, 2H), 5.05-5.16 (m, 2H), 5.74-5.91 (m, 1H), 6.47 (ddd, $J_1$ = 14.7 Hz, $J_2$ = 3.3 Hz, $J_3$ = 1.8 Hz, 1H), 6.58 (dd, $J_1$ = 12.9 Hz, $J_2$ = 3.3 Hz, 1H), 6.79 (d, *J* = 15.0 Hz, 1/2H), 6.87 (d, *J* = 15.0 Hz, 1/2H), 7.25-7.49 (m, 6H), 7.60 (dd, $J_1$ = 15.3 Hz, $J_2$ = 10.8 Hz, 1H); $^{13}$C-NMR ($CDCl_3$, 75.5 MHz): δ 32.0, 33.5, 46.1, 46.5, 49.2, 51.2, 112.0, 112.0, 113.8, 113.9, 114.6, 114.8, 116.5, 117.4, 126.3, 127.1, 127.4, 127.8, 128.4, 128.7, 129.7, 129.8, 134.1, 135.2, 136.9, 137.5, 143.7, 143.8, 151.4, 151.5, 166.2, 166.6; HRMS (ES) calc. for ($M^+$+1) $C_{18}H_{20}NO_2$: 282.1489; found: 282.1483.

**(*E*)-*N*-Benzyl-*N*-(3-butenyl)-3-methyl-2-butenamide (8e)**

Amide **8e** was obtained in 67% yield (yellowish liquid) from (*E*)-2-butenoyl chloride **6a** as a mixture of rotamers following method B. $^1$H-NMR ($CDCl_3$, 300 MHz): δ 1.77 (s, 3/2H), 1.85 (s, 3/2H), 1.97 (d, *J* = 6.0 Hz, 3H), 2.24 (q, *J* = 7.5 Hz, 1H), 2.31 (q, *J* = 7.2 Hz, 1H), 3.29 (t, *J* = 7.4 Hz, 1H), 3.41 (t, *J* = 7.4 Hz, 1H), 4.54 (s, 1H), 4.64 (s, 1H), 4.97-5.07 (m, 2H), 5.62-5.78 (m, 1H), 5.83 (s, 1/2H), 5.90 (s, 1/2H), 7.15-7.36 (m, 5H); $^{13}$C-NMR ($CDCl_3$, 75.5 MHz): δ 20.1, 20.1, 26.1, 26.3, 31.9, 32.7, 44.4, 46.6, 47.6, 51.6, 116.4, 117.0, 117.7, 117.9, 126.4, 127.0, 127.3, 127.8, 128.3, 128.6, 134.3, 135.4, 137.0, 137.7, 147.0, 147.0, 168.2, 168.5; HRMS (ES) calc. for ($M^+$+1) $C_{16}H_{22}NO$: 244.1696; found: 244.1709.

**(*E*)-*N*-Benzyl-*N*-(but-3-en-1-yl)-3-(1*H*-indol-3-yl)acrylamide (8f)**

Amide **8f** was obtained in 67% yield (yellow solid) from (*E*)-3-(1*H*-3-indolyl)acrylic acid **5e** as a mixture of rotamers following method A. m.p. 173-175 ºC. $^{1}$H-NMR ($CDCl_3$, 300 MHz): $\delta$ 2.43 (q, *J* = 7.3 Hz, 2H), 3.52 (t, *J* = 7.7 Hz, 1H), 3.62 (t, *J* = 7.2 Hz, 1H), 4.75 (s, 1H), 4.78 (s, 1H), 5.03-5.19 (m, 2H), 5.77-5.91 (m, 1H), 6.81 (d, *J* = 15.3 Hz, 1/2H), 6.98 (d, *J* = 15.6 Hz, 1/2H), 7.18-7.52 (m, 10H), 7.96-8.07 (m, 1H), 8.76 (br s, 1/2H), 8.84 (br s, 1/2H); $^{13}$C-NMR ($CDCl_3$, 75.5 MHz): $\delta$ 32.5, 33.7, 46.8, 47.1, 49.5, 51.8, 111.8, 111.9, 112.5, 113.1, 114.1, 116.7, 117.5, 120.2, 121.1, 121.2, 123.0, 123.1, 125.2, 126.5, 127.2, 127.5, 128.0, 128.4, 128.6, 128.7, 128.9, 134.5, 135.5, 136.7, 137.1, 137.2, 137.7, 138.0, 167.8, 168.2; HRMS (ES) calc. for ($M^{+}$+1) $C_{22}H_{23}N_2O$: 331.1805; found: 331.1807.

**(*E*)-*N*-Benzyl-*N*-(3-butenyl)-3-(1-methyl-1*H*-3-indolyl)acrylamide (8g)**

Indole **8f** (0.9 mmol) was dissolved in freshly distilled THF (0.05 M) and at 0 ºC NaH (60% dispersion in mineral oil, 1.4 mmol) was added. After 15 min, MeI (1.2 mmol) was added slowly, and the mixture was stirred at rt for 1 hour. The reaction was cooled to 0 ºC, sat aq $NH_4Cl$ (50 mL) was added, and the mixture was extracted with $Et_2O$ (3 x 20 mL). The combined organic layers were washed with 1 M $KHSO_4$ (3 x 50 mL), sat aq $NaHCO_3$ (3 x 20 mL) and brine (20 mL). The organic phase was dried on $Na_2SO_4$, evaporated to dryness, and the crude product was purified by column chromatography (*n*-hexanes:ethyl acetate). Amide **8g** was obtained in 89% yield (yellow solid) as a mixture of rotamers. m.p. 105-107 ºC. $^{1}$H-NMR ($CDCl_3$, 300 MHz): $\delta$ 2.42 (q, *J* = 7.3 Hz, 2H), 3.50 (t, *J* = 7.4 Hz, 1H), 3.60 (t, *J* = 7.5 Hz, 1H), 3.78 (s, 3/2H), 3.82 (s, 3/2H), 4.74 (s, 1H), 4.76 (s, 1H), 5.03-5.18 (m, 2H), 5.77-5.91 (m, 1H), 6.76 (d, *J* = 15.9 Hz, 1/2H), 6.92 (d, *J* = 15.3 Hz, 1/2H), 7.09-7.41 (m, 9H), 7.52 (d, *J* = 8.4 Hz, 1/2H), 7.88 (d, *J* = 7.8 Hz, 1/2H), 7.99 (t, *J* = 16.1 Hz, 1H); $^{13}$C-

NMR ($CDCl_3$, 75.5 MHz): δ 32.0, 32.5, 33.2, 46.3, 46.5, 48.9, 51.3, 109.3, 109.5, 111.4, 111.9, 112.2, 116.1, 116.9, 119.8, 120.4, 120.4, 122.1, 122.2, 125.5, 126.0, 126.7, 127.0, 127.6, 128.0, 128.3, 132.0, 132.3, 134.2, 135.2, 135.8, 136.2, 137.3, 137.4, 137.5, 137.7, 167.2, 167.6; HRMS (ES) calc. for ($M^+$+1) $C_{23}H_{25}N_2O$: 345.1961; found: 345.1965.

**General procedure for the tandem protocol.** A solution of Hoveyda-Grubbs 2nd generation (5 mol %), diene **2** or **8** (3.0 equiv) and alkyne **1** (0.5 mmol) in dry toluene 0.05M was heated at 90 ºC in a sealed tube. The reaction mixture was stirred at this temperature for 48 h. The solvents were then removed under reduced pressure and the crude mixture was purified by flash chromatography in *n*-hexanes:ethyl acetate.

**(4a*R**,8a*S**)-6-Phenyl-3,4,4a,7,8,8a-hexahydro-1*H*-isochromen-1-one (3a).**

Following the general procedure described above, **3a** was obtained in 57% yield as a brown oil. $^1$H-NMR ($CDCl_3$, 300 MHz): δ 1.68-1.87 (m, 2H), 2.00-2.10 (m, 1H), 2.25-2.36 (m, 2H), 2.41-2.53 (m, 1H), 2.75-2.85 (m, 2H), 4.22 (dd, $J_1$ = 6.0 Hz, $J_2$ = 4.5 Hz, 2H), 5.81-5.83 (m, 1H), 7.13-7.29 (m, 5H); $^{13}$C-NMR (CDCl3, 75.5 MHz): δ 24.1, 24.7, 28.5, 32.5, 38.9, 67.3, 124.8, 125.1, 127.3, 128.3, 139.4, 141.2, 173.4; HRMS (ES) calc. for ($M^+$+1) $C_{15}H_{17}O_2$: 229.1223; found: 229.1233.

**(4a*R**,8a*S**)-6-Phenyl-3,4,4a,7,8,8a-hexahydronaphthalen-1(2*H*)-one (3b)**

Following the general procedure described above, **3b** was obtained in 38% yield as a yellow oil. $^1$H-NMR ($CDCl_3$, 300 MHz): δ 1.63-1.73 (m, 1H), 1.77-1.86 (m, 3H), 1.93-2.01 (m, 1H), 2.24-2.44 (m, 4H), 2.56-2.68 (m, 2H), 2.90-2.94 (m, 1H), 5.92-5.94 (m, 1H), 7.28-7.39 (m, 5H); $^{13}$C-NMR ($CDCl_3$, 75.5 MHz): δ 22.8, 23.4, 25.1, 29.9, 38.0, 40.8, 47.5, 125.1, 127.0, 127.1, 128.2, 138.0, 141.6, 212.9; HRMS (ES) calc. for ($M^+$) $C_{16}H_{18}O$: 226.1358; found: 226.1309.

**(4a*R**,8a*S**)-2-Benzyl-6-phenyl-3,4,4a,7,8,8a-hexahydroisoquinolin-1(2*H*)-one (3c).**

Following the general procedure described above, **3c** was obtained in 62% yield as a brown oil. $^1$H-NMR ($CDCl_3$, 300 MHz): δ 1.77-1.88 (m, 1H), 1.95-2.06 (m, 2H), 2.35-2.43 (m, 1H), 2.45-2.59 (m, 2H), 2.73-2.85 (m, 2H), 3.11-3.26 (m, 2H), 4.35 (d, $J$ = 15.0 Hz, 1H), 4.90 (d, $J$ = 15.0 Hz, 1H), 5.92-5.95 (m, 1H), 7.14-7.40 (m, 10H); $^{13}$C-NMR ($CDCl_3$, 75.5 MHz): δ 24.5, 25.5, 27.5, 33.9, 40.0, 45.4, 50.4, 125.1, 125.5, 127.1, 127.1, 127.5, 128.3, 128.5, 137.2, 139.5, 141.6, 171.8; HRMS (ES) calc. for ($M^+$+1) $C_{22}H_{24}NO$: 318.1852; found: 318.1866

**(4a*R**,8a*S**)-2-Benzyl-6-(4-fluorophenyl)-3,4,4a,7,8,8a-hexahydroisoquinolin-1(2*H*)-one (3d)**

Following the general procedure described above, **3e** was obtained in 45% yield as a yellow oil. $^1$H-NMR ($CDCl_3$, 300 MHz): δ 1.76-1.86 (m, 1H), 1.94-2.04 (m, 2H), 2.33-2.52 (m, 3H), 2.72-2.82 (m, 2H), 3.10-3.24 (m, 2H), 4.31 (d, $J$ = 14.7 Hz, 1H), 4.92 (d, $J$ = 14.7 Hz, 1H), 5.86-5.87 (m, 1H), 6.98-7.03 (m, 2H), 7.12-7.21 (m, 5H), 7.29-7.34 (m, 2H); $^{13}$C-NMR ($CDCl_3$, 75.5 MHz): δ 24.5, 25.7, 27.5, 33.9, 39.9, 45.4, 50.4, 115.0 (d, $^2J_{CF}$ = 21.3 Hz), 125.4, 126.7 (d, $^3J_{CF}$ = 7.8 Hz), 127.1, 127.5, 128.5, 137.2, 137.7 (d, $^4J_{CF}$ = 2.9 Hz), 138.6, 162.1 (d, $^1J_{CF}$ = 246.0 Hz), 171.7; $^{19}$F-NMR ($CDCl_3$, 282 MHz): δ -116.1; HRMS (ES) calc. for ($M^+$+1) $C_{22}H_{23}NOF$: 336.1758; found: 336.1749.

**(4a*R**,8a*S**)-2-Benzyl-6-(4-methoxyphenyl)-3,4,4a,7,8,8a-hexahydroisoquinolin-1(2*H*)-one (3e).**

Following the general procedure described above, **3d** was obtained in 35% yield as a yellow oil. $^1$H-NMR ($CDCl_3$, 300 MHz): δ 1.76-1.86 (m, 1H), 1.93-2.04 (m, 2H), 2.30-2.49 (m, 3H), 2.71-2.82 (m, 2H), 3.09-3.25 (m, 2H), 3.82 (s, 3H), 4.34 (d, $J$ =

14.7 Hz, 1H), 4.88 (d, *J* = 14.7 Hz, 1H), 5.84-5.85 (m, 1H), 6.86 (d, $J_o$ = 8.7 Hz, 2H), 7.12-7.21 (m, 5H), 7.30-7.33 (m, 2H); $^{13}$C-NMR ($CDCl_3$, 75.5 MHz): δ 24.6, 25.6, 27.6, 33.9, 40.1, 45.4, 50.4, 55.3, 113.6, 123.9, 126.2, 127.1, 127.5, 128.5, 129.9, 134.2, 137.2, 149.7, 166.4; HRMS (ES) calc. for ($M^+$+1) $C_{23}H_{26}NO_2$: 348.1958; found: 348.1943.

**(3a*R**,7a*S**)-2-Benzyl-5-phenyl-2,3,3a,6,7,7a-hexahydro-1*H*-isoindol-1-one (3f)**

Following the general procedure described above, **3f** was obtained in 50% yield as a brown oil. $^1$H-NMR ($CDCl_3$, 300 MHz): δ 1.87-1.98 (m, 1H), 2.24 (ddd, $J_1$ = 13.2 Hz, $J_2$ = 10.5 Hz, $J_3$ = 5.4 Hz, 1H), 2.35-2.44 (m, 1H), 2.45-2.57 (m, 1H), 2.81 (dt, $J_1$ = 7.5 Hz, $J_2$ = 5.1 Hz, 1H), 2.97 (dd, $J_1$ = 9.6 Hz, $J_2$ = 3.3 Hz, 1H), 3.02-3.10 (m, 1H), 3.51 (dd, $J_1$ = 9.3 Hz, $J_2$ = 7.2 Hz, 1H), 4.38 (d, *J* = 15.0 Hz, 1H), 4.60 (d, *J* = 15.0 Hz, 1H), 5.83-5.85 (m, 1H), 7.14-7.34 (m, 10H); $^{13}$C-NMR ($CDCl_3$, 75.5 MHz): δ 21.4, 24.4, 32.7, 40.1, 46.3, 51.4, 124.1, 125.2, 127.2, 127.4, 127.7, 128.3, 128.6, 136.4, 139.9, 141.8, 175.4; HRMS (ES) calc. for ($M^+$+1) $C_{21}H_{22}NO$: 304.1696; found: 304.1702.

**(5a*R**,9a*S**)-2-Benzyl-7-phenyl-2,3,4,5,5a,8,9,9a-octahydro-1*H*-benzo[*c*]azepin-1-one (3g)**

Following the general procedure described above, **3g** was obtained in 44% yield as a yellowish oil. $^1$H-NMR ($CDCl_3$, 300 MHz): δ 1.31 (q, *J* = 10.5 Hz, 2H), 1.68-1.74 (m, 1H), 1.95-2.56 (m, 7H), 3.17 (dd, $J_1$ = 15.3 Hz, $J_2$ = 6.0 Hz, 1H), 3.45 (dd, $J_1$ = 15.3 Hz, $J_2$ = 10.5 Hz, 1H), 4.44 (d, *J* = 14.7 Hz, 1H), 4.75 (d, *J* = 14.4 Hz, 1H), 5.74 (s, 1H), 7.15-7.35 (m, 10H); $^{13}$C-NMR ($CDCl_3$, 75.5 MHz): δ 25.4, 26.4, 28.0, 37.4, 38.2, 44.2, 48.0, 51.3, 125.1, 126.9, 127.3, 128.3, 128.3, 128.5, 128.6, 136.7, 138.1, 141.8, 176.5; HRMS (ES) calc. for ($M^+$+1) $C_{23}H_{26}NO$: 332.2009; found: 332.2012.

**(8*R**,8a*S**)-2-Benzyl-8-methyl-6-phenyl-3,4,4a,7,8,8a-hexahydroisoquinolin-1(2*H*)-one (10a)**

Following the general procedure described above, **10a** was obtained in 85% yield (*endo*:*exo* 93:7) as a yellow oil. ***endo* isomer:** $^{1}$H-NMR ($CDCl_3$, 300 MHz): δ 1.12 (d, $J$ = 7.2 Hz, 3H), 1.91-2.05 (m, 2H), 2.07-2.15 (m, 1H), 2.45 (t, $J$ = 5.0 Hz, 1H), 2.60-2.70 (m, 1H), 2.86 (br s, 1H), 2.95-3.10 (m, 2H), 3.22 (td, $J_1$ = 12.0 Hz, $J_2$ = 5.4 Hz, 1H), 4.27 (d, $J$ = 15.0 Hz, 1H), 4.92 (d, $J$ = 15.0 Hz, 1H), 5.87 (s, 1H), 7.08-7.17 (m, 5H), 7.23-7.38 (m, 5H); $^{13}$C-NMR ($CDCl_3$, 75.5 MHz): δ 18.8, 27.5, 28.0, 29.9, 31.2, 44.6, 45.1, 50.6, 124.3, 125.2, 127.0, 127.1, 127.3, 128.3, 128.5, 137.2, 138.4, 141.9, 171.2; HRMS (ES) calc. for ($M^{+}$+1) $C_{23}H_{26}NO$: 332.2009; found: 332.1995.

***N*-Benzyl-*N*-[(*Z*)-5-phenylhexa-3,5-dien-1-yl]cinnamamide (9b-cis)**

Intermediate **9b-cis** was detected as a mixture of rotamers in 15% yield. $^{1}$H-NMR ($CDCl_3$, 300 MHz): δ 2.42-2.50 (m, 2H), 3.39 (t, $J$ = 7.4 Hz, 1H), 3.53 (t, $J$ = 7.4 Hz, 1H), 4.53 (s, 1H), 4.66 (s, 1H), 5.13 (s, 1/2H), 5.15 (s, 1/2H), 5.55 (s, 1H), 5.66 (ddd, $J_1 = J_{cis}$ = 11.4 Hz, $J_2$ = 7.5 Hz, $J_3$ = 7.5 Hz, 1/2H), 5.77 (ddd, $J_1 = J_{cis}$ = 11.4 Hz, $J_2$ = 7.5 Hz, $J_3$ = 7.5 Hz, 1/2H), 6.26 (d, $J_{cis}$ = 10.7 Hz, 1/2H), 6.30 (d, $J_{cis}$ = 10.7 Hz, 1/2H), 6.75 (d, $J$ = 15.3 Hz, 1/2H), 6.84 (d, $J$ = 15.3 Hz, 1/2H), 7.15-7.46 (m, 15H), 7.75 (d, $J$ = 15.3 Hz, 1/2H), 7.77 (d, $J$ = 15.3 Hz, 1/2H); $^{13}$C-NMR ($CDCl_3$, 75.5 MHz): δ 27.0, 28.3, 46.1, 46.9, 49.1, 51.0, 115.0, 115.1, 117.1, 117.5, 126.3, 126.4, 126.5, 126.9, 127.3, 127.6, 127.8, 127.8, 128.0, 128.3, 128.4, 128.5, 128.5, 128.7, 128.7, 128.8, 129.6, 130.3, 131.0, 132.1, 135.2, 135.2, 137.0, 137.5, 140.0, 140.9, 143.1, 143.2, 144.0, 144.1, 166.5, 166.9; HRMS (ES) calc. for ($M^{+}$+1) $C_{28}H_{28}NO$: 394.2165; found: 394.2150.

**(8*R**,8a*R**)-2-Benzyl-6,8-diphenyl-3,4,4a,7,8,8a-hexahydroisoquinolin-1(2*H*)-one (10b)**

Following the general procedure described above, **10b** was obtained in 78% overall yield (yellow oil) as a separable mixture of isomers (*exo*:*endo* 47:53). ***exo* isomer:** $^{1}$H-NMR ($CDCl_3$, 300 MHz): δ 1.67-1.80 (m, 1H), 2.10-2.20 (m, 1H), 2.53-2.84 (m, 4H), 3.21-3.44 (m, 3H), 4.33 (d, $J$ = 14.7 Hz, 1H), 4.67 (d, $J$ = 14.7 Hz, 1H), 6.01 (s, 1H), 7.18-7.40 (m, 15H); $^{13}$C-NMR ($CDCl_3$, 75.5 MHz): δ 29.4, 37.2, 38.9, 41.7, 44.5, 46.9, 49.5, 125.1, 125.7, 126.0, 126.8, 127.2, 127.3, 128.1, 128.3, 128.4, 128.5, 136.9, 137.6, 140.6, 147.5, 171.6; HRMS (ES) calc. for ($M^{+}$+1) $C_{28}H_{28}NO$: 394.2165; found: 394.2145. ***endo* isomer:** $^{1}$H-NMR ($CDCl_3$, 300 MHz): δ 1.89-1.95 (m, 2H), 2.47 (br s, 1H), 2.71-2.80 (m, 2H), 2.92-3.02 (m, 1H), 3.08-3.14 (m, 1H), 3.22-3.32 (m, 1H), 4.26-4.31 (m, 2H), 5.02 (d, $J$ = 15.0 Hz, 1H), 6.02 (s, 1H), 7.12-7.52 (m, 15H); $^{13}$C-NMR ($CDCl_3$, 75.5 MHz): δ 27.4, 27.7, 29.2, 38.3, 44.7, 46.5, 50.6, 125.3, 125.8, 126.1, 127.0, 127.2, 127.4, 127.4, 128.3, 128.4, 128.5, 137.0, 139.5, 141.2, 144.5, 170.6; HRMS (ES) calc. for ($M^{+}$+1) $C_{28}H_{28}NO$: 394.2165; found: 394.2186.

**(8*R**,8a*R**)-2-Benzyl-8-(naphthalen-2-yl)-6-phenyl-3,4,4a,7,8,8a-hexahydroisoquinolin-1(2*H*)-one (10c)**

Following the general procedure described above, **10c** was obtained in 47% overall yield (yellow oil) as a separable mixture of isomers (*exo*:*endo* 34:66). ***exo* isomer:** $^{1}$H-NMR ($CDCl_3$, 300 MHz): δ 1.87-1.93 (m, 2H), 2.46 (br s, 1H), 2.85-2.91 (m, 2H), 3.01-3.14 (m, 2H), 3.28 (td, $J_1$ = 12.0 Hz, $J_2$ = 6.9 Hz, 1H), 4.30 (d, $J$ = 15.0 Hz, 1H), 4.46-4.48 (m, 1H), 5.05 (d, $J$ = 15.0 Hz, 1H), 6.04 (s, 1H), 7.15-7.20 (m, 5H), 7.36-7.49 (m, 5H), 7.53-7.58 (m, 3H), 7.75-7.84 (m, 4H); $^{13}$C-NMR ($CDCl_3$, 75.5 MHz): δ 27.4, 27.8, 29.3, 38.5, 44.7, 46.3, 50.6, 125.3, 125.4, 125.8, 126.3, 127.0, 127.2, 127.4, 127.5, 127.7, 127.9, 128.5, 128.5, 132.0, 133.3, 137.0, 139.4, 141.2, 141.8,

170.5; HRMS (ES) calc. for ($M^+$+1) $C_{32}H_{30}NO$: 444.2322; found: 444.2338. ***endo* isomer:** $^1$H-NMR ($CDCl_3$, 300 MHz): δ 1.69-1.86 (m, 1H), 2.10-2.24 (m, 1H), 2.63-2.77 (m, 2H), 2.82-2.92 (m, 2H), 3.29 (dt, $J_1$ = 12.9 Hz, $J_2$ = 7.8 Hz, 1H), 3.39-3.53 (m, 2H), 4.35 (d, *J* = 14.7 Hz, 1H), 4.64 (d, *J* = 14.7 Hz, 1H), 6.05 (s, 1H), 7.18-7.54 (m, 13H), 7.76-7.87 (m, 4H); $^{13}$C-NMR ($CDCl_3$, 75.5 MHz): δ 29.5, 37.2, 38.9, 41.9, 44.6, 47.0, 49.5, 124.8, 125.1, 125.6, 125.7, 126.1, 127.2, 127.3, 127.6, 127.7, 127.9, 128.1, 128.3, 128.5, 132.1, 133.8, 137.0, 137.6, 140.5, 145.1, 171.6; HRMS (ES) calc. for ($M^+$+1) $C_{32}H_{30}NO$: 444.2322; found: 444.2337.

**(8*R**,8a*R**)-2-Benzyl-8-(furan-2-yl)-6-phenyl-3,4,4a,7,8,8a-hexahydroisoquinolin-1(2*H*)-one (10d)**

Following the general procedure described above, **10d** was obtained in 68% yield (yellow oil) as an inseparable mixture of isomers (*exo*:*endo* 28:72). $^1$H-NMR ($CDCl_3$, 300 MHz): δ 1.60-1.71 (m, 1/2H), 1.77-1.85 (m, 1H), 1.87-2.07 (m, 3/2H), 2.49-2.70 (m, 4H), 2.75-2.85 (m, 1H), 2.99-3.04 (m, 2H), 3.10-3.20 (m, 3/2H), 3.22-3.38 (m, 1H), 4.18-4.22 (m, 2H), 4.37 (d, *J* = 14.4 Hz, 1/2H), 4.57 (d, *J* = 14.7 Hz, 1/2H), 4.89 (d, *J* = 15.0 Hz, 1H), 5.77 (br s, 1H), 5.87-5.88 (m, 1/2H), 5.95-5.97 (m, 1H), 6.04-6.06 (m, 1/2H), 6.19 (dd, $J_1$ = 3.0 Hz, $J_2$ = 1.8 Hz, 1H), 6.30 (dd, $J_1$ = 3.3 Hz, $J_2$ = 1.8 Hz, 1/2H), 7.30-7.34 (m, 16.5H); $^{13}$C-NMR ($CDCl_3$, 75.5 MHz): δ 27.2, 27.4, 29.4, 30.2, 33.6, 35.2, 35.7, 37.0, 43.0, 44.6, 44.9, 46.1, 49.6, 50.6, 103.6, 105.2, 110.0, 110.4, 125.0, 125.1, 125.2, 126.0, 127.0, 127.2, 127.2, 127.3, 127.3, 128.1, 128.3, 128.4, 128.5, 128.5, 136.6, 137.0, 137.5, 138.4, 140.1, 140.5, 140.9, 141.5, 157.5, 159.1, 170.1, 170.9; HRMS (ES) calc. for ($M^+$+1) $C_{26}H_{26}NO_2$: 384.1958; found: 384.1958.

**(8a*R**)-2-Benzyl-8,8-dimethyl-6-phenyl-3,4,4a,7,8,8a-hexahydroisoquinolin-1(2*H*)-one (10e)**

Following the general procedure described above, **10e** was obtained in 25% yield (brown oil) as a separable mixture of isomers (*exo*:*endo* 23:77). ***exo* isomer:** $^{1}$H-NMR ($CDCl_3$, 300 MHz): δ 1.02 (s, 3H), 1.49 (s, 3H), 1.53-1.67 (m, 1H), 1.97-2.13 (m, 3H), 2.36-2.51 (m, 2H), 3.19-3.24 (m, 2H), 4.44 (d, $J$ = 14.7 Hz, 1H), 4.59 (d, $J$ = 14.7 Hz, 1H), 5.79 (t, $J$ = 2.3 Hz, 1H), 7.15-7.34 (m, 10H); $^{13}$C-NMR ($CDCl_3$, 75.5 MHz): δ 21.3, 29.7, 31.1, 32.5, 34.6, 45.3, 45.9, 49.8, 51.7, 125.1, 125.6, 127.1, 127.2, 127.8, 128.3, 128.5, 136.2, 137.7, 141.3, 171.6; HRMS (ES) calc. for ($M^{+}$+1) $C_{24}H_{28}NO$: 346.2165; found: 346.2163. ***endo* isomer:** $^{1}$H-NMR ($CDCl_3$, 300 MHz): δ 1.07 (s, 3H), 1.45 (s, 3H), 1.83-1.91 (m, 2H), 2.08 (d, $J$ = 17.4 Hz, 1H), 2.26 (d, $J$ = 6.0 Hz, 1H), 2.50 (dt, $J_1$ = 17.4 Hz, $J_2$ = 2.7 Hz, 1H), 2.89 (br s, 1H), 3.01 (dt, $J_1$ = 12.0 Hz, $J_2$ = 4.5 Hz, 1H), 3.12-3.21 (m, 1H), 4.14 (d, $J$ = 15.0 Hz, 1H), 4.84 (d, $J$ = 15.0 Hz, 1H), 5.82 (s, 1H), 7.01-7.08 (m, 4H), 7.18-7.33 (m, 6H); $^{13}$C-NMR ($CDCl_3$, 75.5 MHz): δ 27.7, 29.4, 29.7, 33.4, 34.0, 39.1, 45.2, 49.0, 50.6, 124.8, 125.3, 127.0, 127.1, 127.4, 128.3, 128.5, 137.4, 138.9, 141.8, 171.0; HRMS (ES) calc. for ($M^{+}$+1) $C_{24}H_{28}NO$: 346.2165; found: 346.2167.

**(8*R**,8a*R**)-2-Benzyl-8-(1-methyl-1*H*-indol-3-yl)-6-phenyl-3,4,4a,7,8,8a-hexahydroisoquinolin-1(2*H*)-one (10f)**

Following the general procedure described above, **10f** was obtained in 33% yield (yellow oil) as a separable mixture of isomers (*exo*:*endo* 50:50). ***exo* isomer:** $^{1}$H-NMR ($CDCl_3$, 300 MHz): δ 1.86-1.91 (m, 2H), 2.58 (br s, 1H), 2.73 (d, $J$ = 18.0 Hz, 1H), 2.96-3.12 (m, 3H), 3.21-3.31 (m, 1H), 3.70 (s, 3H), 4.30 (d, $J$ = 15.0 Hz, 1H), 4.59-4.62 (m, 1H), 5.04 (d, $J$ = 15.0 Hz, 1H), 5.97 (s, 1H), 6.83 (s, 1H), 7.13-7.18 (m, 5H), 7.23-7.45 (m, 6H), 7.49-7.53 (m, 2H), 7.83 (dt, $J_1$ = 7.8 Hz, $J_2$ = 1.1 Hz, 1H); $^{13}$C-

NMR ($CDCl_3$, 75.5 MHz): δ 27.5, 29.3, 30.0, 30.5, 32.6, 44.0, 44.8, 50.6, 109.1, 117.2, 118.7, 119.2, 121.5, 125.2, 125.2, 126.4, 127.0, 127.0, 127.3, 127.4, 128.4, 128.5, 136.8, 137.2, 139.2, 141.5, 171.0; HRMS (ES) calc. for ($M^+$+1) $C_{31}H_{31}N_2O$: 447.2431; found: 447.2437. ***endo* isomer:** $^1$H-NMR ($CDCl_3$, 300 MHz): δ 1.68-1.81 (m, 1H), 2.12-2.23 (m, 1H), 2.61-2.73 (m 1H), 2.84-2.92 (m, 3H), 3.22-3.31 (m, 1H), 3.39-3.48 (m, 1H), 3.66-3.75 (m, 1H), 3.77 (s, 3H), 4.31 (d, *J* = 14.7 Hz, 1H), 4.71 (d, *J* = 14.7 Hz, 1H), 6.06 (d, *J* = 2.1 Hz, 1H), 6.99 (s, 1H), 7.04-7.10 (m, 1H), 7.17-7.34 (m, 10H), 7.37-7.41 (m, 2H), 7.71 (d, *J* = 8.1 Hz, 1H); $^{13}$C-NMR ($CDCl_3$, 75.5 MHz): δ 29.4, 32.7, 32.8, 37.0, 37.2, 44.2, 46.7, 49.5, 109.5, 118.3, 119.5, 120.1, 121.0, 125.2, 125.6, 126.1, 126.6, 127.1, 127.2, 128.0, 128.3, 128.5, 137.2, 137.8, 140.8, 172.3; HRMS (ES) calc. for ($M^+$+1) $C_{31}H_{31}N_2O$: 447.2431; found: 447.2439.

# References


1. D´Annibale, A.; Ciaralli, L. *J. Org. Chem.* **2007**, *72*, 6067.

2. Qian, M.; Covey, D. F. *Adv. Synth. Cat.* **2010**, *352*, 2057.

3. Takeaki, N.; Yuko, H.; Okiko, M.; Ichiya, N. *J. Chem. Soc., Perkin Trans. 1,* **1995**, 19.

4. Petersen, M. T.; Nielsen, T. E. *Org. Lett.* **2013**, *15*, 1986.
